# Intracellular Long-Chain Acyl CoAs Activate TRPV1 Channels

**DOI:** 10.1371/journal.pone.0096597

**Published:** 2014-05-05

**Authors:** Yi Yu, Chris R. J. Carter, Nermeen Youssef, Jason R. B. Dyck, Peter E. Light

**Affiliations:** 1 Department of Pharmacology, Alberta Diabetes Institute, Faculty of Medicine and Dentistry, University of Alberta, Edmonton, Alberta, Canada; 2 Department of Pediatrics, Faculty of Medicine and Dentistry, University of Alberta, Edmonton, Alberta, Canada; Sackler Medical School, Tel Aviv University, Israel

## Abstract

TRPV1 channels are an important class of membrane proteins that play an integral role in the regulation of intracellular cations such as calcium in many different tissue types. The anionic phospholipid phosphatidylinositol 4,5-bisphosphate (PIP_2_) is a known positive modulator of TRPV1 channels and the negatively charged phosphate groups interact with several basic amino acid residues in the proximal C-terminal TRP domain of the TRPV1 channel. We and other groups have shown that physiological sub-micromolar levels of long-chain acyl CoAs (LC-CoAs), another ubiquitous anionic lipid, can also act as positive modulators of ion channels and exchangers. Therefore, we investigated whether TRPV1 channel activity is similarly regulated by LC-CoAs. Our results show that LC-CoAs are potent activators of the TRPV1 channel and interact with the same PIP_2_-binding residues in TRPV1. In contrast to PIP_2_, LC-CoA modulation of TRPV1 is independent of Ca^2+^_i_, acting in an acyl side-chain saturation and chain-length dependent manner. Elevation of LC-CoAs in intact Jurkat T-cells leads to significant increases in agonist-induced Ca^2+^_i_ levels. Our novel findings indicate that LC-CoAs represent a new fundamental mechanism for regulation of TRPV1 channel activity that may play a role in diverse cell types under physiological and pathophysiological conditions that alter fatty acid transport and metabolism such as obesity and diabetes.

## Introduction

Calcium ions (Ca^2+^) play a crucial role in a vast number of cellular processes [1]. Our knowledge of the trans-membrane Ca^2+^ transport proteins has increased with the discovery of a novel superfamily of trans-membrane ion channels termed “transient receptor potential” or TRP. TRP channels contribute to alterations in the concentration cytosolic free Ca^2+^ ([Ca^2+^]_i_) via two mechanisms. Certain TRP channel subtypes are Ca^2+^ permeable and directly transport Ca^2+^ into the cell across the plasma membrane, while Ca^2+^ impermeable TRP channels can alter membrane potential and therefore modulate the driving force for Ca^2+^ through other transport mechanisms [2]. Mutations in several TRP proteins underlie human diseases in relation to dysfunction in Ca^2+^ signalling [3]. Therefore, physiological or pathophysiological changes in TRP channel activity, by whatever mechanism, may also lead to altered in cellular Ca^2+^ signalling/handling, resulting in dysfunction at the level of the cell, organ and whole organism.

TRP channels are ubiquitously expressed in many cell types throughout the body and their principal physiological function is thought to be as sensors for a wide range of physical and chemical stimuli [4]. However, recent work has elucidated a role of TRP channels in the pathophysiology of certain diseases including autoimmune and metabolic diseases such as multiple sclerosis (MS), type 1 and type 2 diabetes, atherosclerosis, obesity, dyslipidemia and metabolic syndrome [5]. While much is known regarding their molecular biology, a comprehensive understanding of factors that regulate these channels is currently incomplete. Given the documented importance of TRP channels in metabolic disorders, the characterization of intrinsic regulation of TRP channel activity via endogenous metabolic and signalling pathways is highly relevant. Elucidation of these regulatory processes has the potential to provide crucial insights into the mechanisms by which TRP channels are modulated in health and disease in a variety of cells, organs and the organism as a whole. One area of current interest is the role of lipid mediators in regulating ionic homeostasis via the modulation of ion channels and transporters. For example, the anionic phospholipid phosphatidylinositol 4, 5-bisphosphate (PIP_2_) modulates the activity of multiple members of the TRP channel superfamily including TRPV1-6, TRPM4-8 and TRPC6 and 7 [6], [7]. TRPV1 was the first TRP channel to be identified and cloned and is a Ca^2+^ permeable member of the vanilloid family of TRP channels activated by capsaicin, temperature and acidic pH. Subsequent studies have revealed putative PIP_2_-interacting domains in the polybasic proximal C-terminal region of TRPV1 [8]. In addition, polyunsaturated fatty acids, their metabolites and lysophosphatidic acid (PLA) are also known to modulate TRPV1channel function [9]–[11].

Interestingly, intracellular levels of the anionic long chain acyl CoA esters (LC-CoAs) are increased in many pathophysiological conditions including those mentioned above, resulting in alterations of metabolic enzyme activity, gene transcription and the immune mediated inflammatory response [3]. Similar to PIP_2_, LC-CoAs are comprised of a hydrophobic tail with a negatively charged head group (CoA). Our group and others have shown that LC-CoAs have a direct and potent stimulatory effect on the ATP-sensitive potassium (K_ATP_) channel [12]–[14] and that PIP_2_ and LC-CoAs possess a similar molecular mechanism of action via interaction with intracellular positively charged basic regions of the K_ATP_ channel [15], [16]. Additional work in our laboratory also shows that LC-acyl CoAs and PIP_2_ modulate the sodium-calcium exchanger via interaction with common basic residues [17].

Therefore, in this current study we investigated whether LC-CoAs, like PIP_2_, regulate TRPV1 activity by characterizing the effects of physiological intracellular concentrations of common dietary LC-CoAs on recombinant TRPV1 channel activity. We also determined the effects of intracellular LC-CoA elevation on TRPV1 channel-mediated intracellular Ca^2+^ accumulation in intact cell models. Finally, we investigated the role of known PIP_2_ interacting amino acid residues in the TRPV1 channel to elucidate the molecular interactions responsible for LC-CoA modulation of TRPV1 channel function.

Our results demonstrate that sub-micromolar physiological levels LC-CoAs are potent positive modulators of TRPV1 channel activity and act via a similar, but not identical, molecular mechanism to PIP_2_.

## Results

### Palmitoyl CoA rescues TRPV1 from the desensitized state

**Figure 1:**
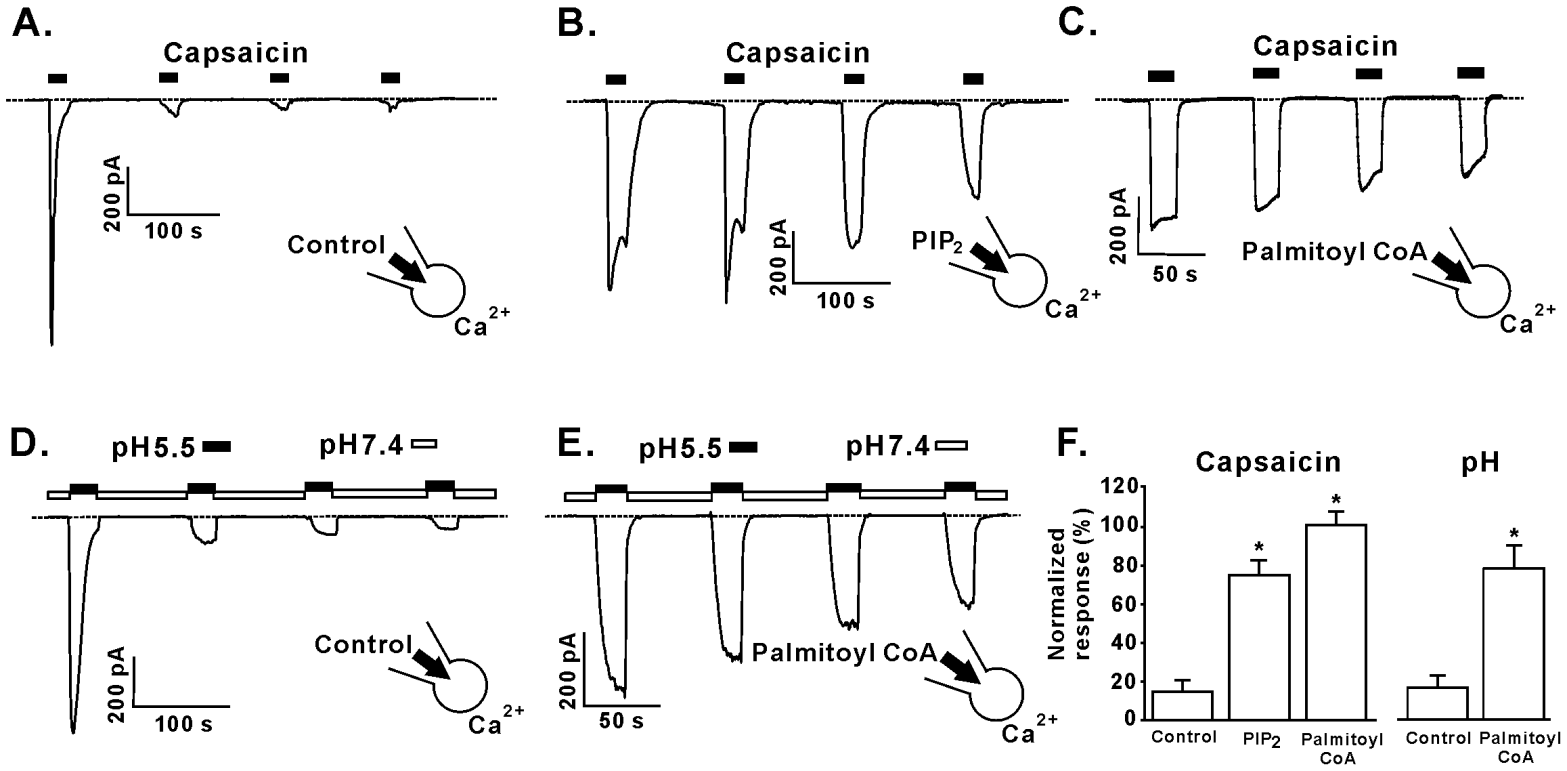
The effect of palmitoyl CoA on TRPV1 currents. (A) Representative whole-cell recordings elicited by 1 µM capsaicin (n = 5) in the presence of (B) 25 µM PIP_2_ (n = 12) or (C) 1 µM palmitoyl CoA (n = 9). (D) Representative whole-cell recordings elicited by acidic pH (pH = 5.5, n = 5) in the presence of (E) 1 µM palmitoyl CoA (n = 5). (F) Grouped data of the effects of PIP_2_ and palmitoyl CoA on TRPV1 currents. *P<0.05. Dashed line denotes zero current level.

Palmitoyl CoA is the intracellular CoA ester of palmitate, a 16 carbon saturated fatty acid and was used in these experiments to represent a typical dietary saturated fatty acid that is abundant in Western diets [18], [19]. To determine whether intracellular palmitoyl CoA can act as an endogenous regulator of TRPV1 channel function we performed whole-cell patch clamp recording on tsA201 cells expressing recombinant TRPV1 channel. PIP_2_ or palmitoyl CoA (1 µM) were added to the intracellular solution and whole-cell currents were recorded >5 minutes after patch-rupture. Extracellular application of the TRPV1 agonist capsaicin (1 µM) activated inward currents that displayed characteristic desensitization of TRPV1 following repeated agonist application (Fig. 1A). PIP_2_ (Fig. 1B) or palmitoyl CoA (Fig. 1C) resulted in a marked reduction of TRPV1 desensitization in response to repeated capsaicin exposures. In the presence of palmitoyl CoA, the second capsaicin-elicited current response was 101.2±6.3% of the first response, compared to 15.1±4.2% in the absence of palmitoyl CoA (P<0.01). PIP_2_ also increased the magnitude of the second capsaicin-elicited current (75.5±5.7%) (P<0.01 versus control), although the PIP_2_ effect was not as great as that of palmitoyl CoA (P<0.05, Fig. 1F).

Next, we investigated the role of intracellular LC-CoAs on TRPV1 channel function using protons as a TRPV1 agonist that activates channels through an alternate molecular mechanism to capsaicin [20], [21]. TRPV1 currents were elicited changing the pH of the superfusate from 7.4 to 5.5 (Fig. 1D). The magnitude of the proton-activated TRPV1 responses were not significantly different than those evoked by capsaicin. Current densities after the first application of capsaicin or pH5.5 were very similar (492.9±32.8pA/pF and 408.4±57.0pA/pF, respectively, P>0.05). Furthermore, we determined the effect of 1 µM palmitoyl CoA proton-mediated TRPV1 desensitization by repeated switching of solution pH from 7.4 to 5.5 (Fig. 1E). In a similar manner to capsaicin-elicited currents, inclusion of palmitoyl CoA in the recording pipette significantly reduced the desensitization induced by protons (second current response was 78.9±12.0% of the first response vs 17.3±6.4%, for control (P = 0.01, Fig. 1F). While capsaicin activates TRPV1 at an intracellular binding site [20], extracellular acidic residues on the TRPV1 channel may play a role in proton activation [21], we did not observe any differences in the extent of palmitoyl CoA rescue of either capsaicin- or proton-evoked desensitization (second agonist response: 101.2±6.3 vs. 78.9±12%, P>0.05, Fig. 1F).

### Ca^2+^ and LC-CoA modulation of TRPV1 activity

**Figure 2:**
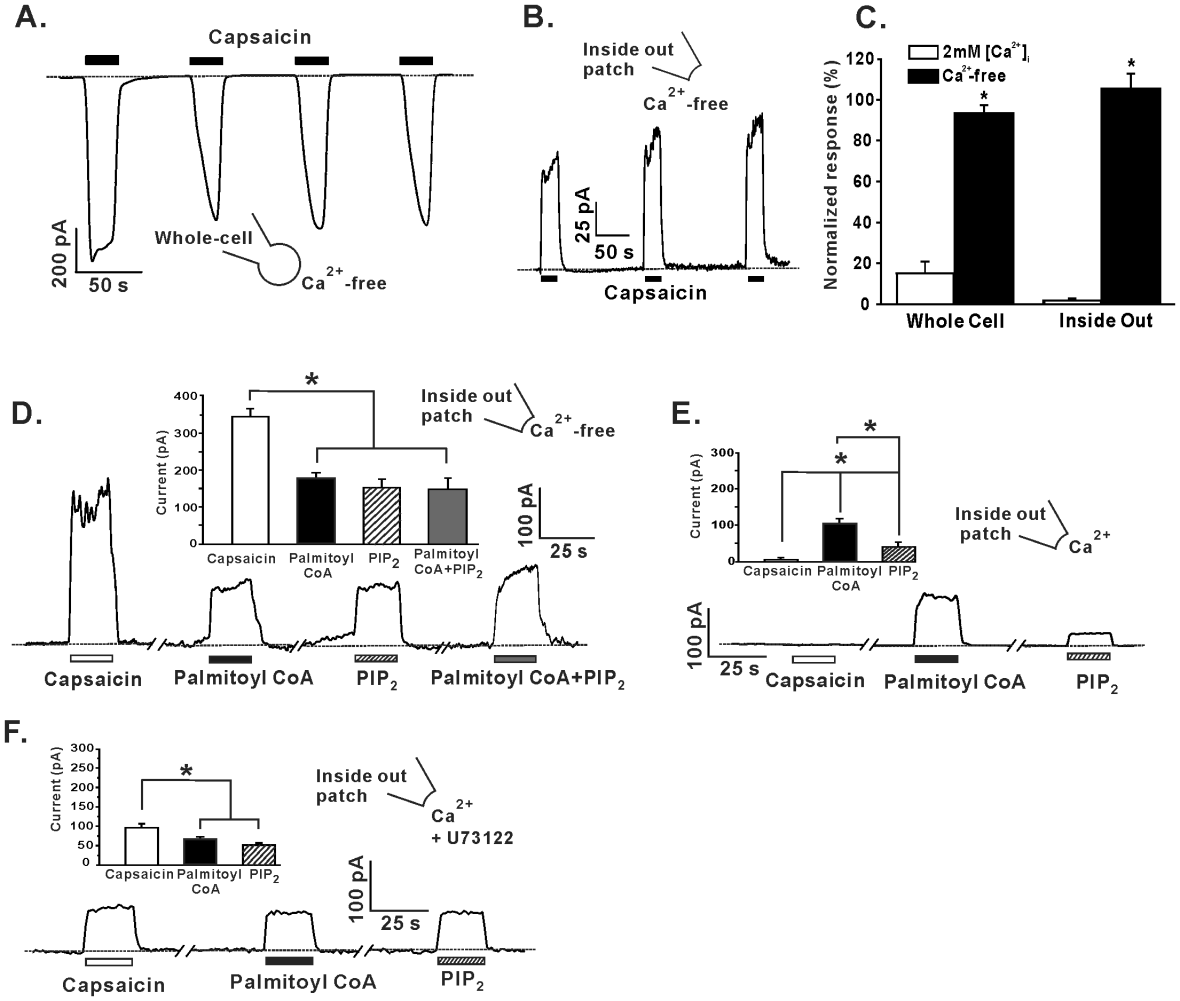
The role of Ca^2+^ in PIP_2_ and palmitoyl CoA regulation of TRPV1 currents. (A) Representative whole-cell recordings (n = 7) and (B) inside-out recording (n = 5) of currents elicited by 1 µM capsaicin in the absence of Ca^2+^. (C) Grouped data of the effect of Ca^2+^ on the desensitization of TRPV1 currents. (D) Representative inside-out recordings and histograms of the effect of 1 µM palmitoyl CoA (n = 7), 25 µM PIP_2_ (n = 4) or 1 µM palmitoyl CoA combined with 25 µM PIP_2_ (n = 9) on currents elicited by 1 µM capsaicin (n = 9) in the absence of Ca^2+^. (E) Representative inside-out recordings and grouped data of the application of 1 µM capsaicin (n = 6), 1 µM palmitoyl CoA (n = 8) or 25 µM PIP_2_ (n = 8) in the presence of 2 mM Ca^2+^. (F) Representative inside-out recordings and grouped data of the effect of 1 µM capsaicin (n = 12), 1 µM palmitoyl CoA (n = 11) or 25 µM PIP_2_ (n = 10) in the presence of 2 mMCa^2+^ and 2 µM U73122. *P<0.05. Dashed line denotes zero current level.

TRPV1 channel desensitization is Ca^2+^_i_-dependent and the underlying mechanism is thought to involve depletion of the open channel stabilizing anionic lipid PIP_2_ from the membrane via its cleavage by the Ca^2+^-dependent enzyme phospholipase C (PLC) [22]. TRPV1 currents elicited by 1 µM capsaicin showed little desensitization following repeated agonist exposure in the absence of extracellular or intracellular Ca^2+^ (Fig. 2A–C, 93.3±3.9%, and 105.3±7.5% respectively, vs. 78.9±12.0% for control). In the absence of Ca^2+^_i_, capsaicin-elicited currents could be rescued to ∼50% by the application of 1 µM palmitoyl CoA (177.1±15.4pA), 25 µM PIP_2_ (153.7±22) or the combination of 1 µM palmitoyl CoA and 25 µM PIP_2_ (148.6±29.2pA) versus control (346.9±20.1pA), P<0.05 (Fig. 2D). However, in the presence of 2 mM Ca^2+^, 1 µM capsaicin elicited currents of much smaller magnitude (6.2±1.9pA) compared to subsequent currents in the presence of 1 µM palmitoyl CoA (103.5±13.9pA) or 25 µM PIP_2_ (39.9±12.6pA) (P<0.05) (Fig. 2E). Furthermore, palmitoyl CoA had a greater ability to rescue the capsaicin-elicited current than PIP_2_ (P<0.05). These data suggest that in the presence of 2 mM Ca^2+^, PLC activity is increased, resulting in the cleavage and depletion of PIP_2_ from the membrane. To test this concept, the PLC inhibitor U73122 (2 µM) was able to rescue capsaicin-elicited currents in the presence of 2 mM Ca^2+^ (98.0±8.8pA) to levels not significantly different (P>0.05) from subsequent currents in the presence of 1 µM palmitoyl CoA (66.5±6.2pA) or PIP_2_ (51.6±5.3pA) (Fig. 2F). In the presence of U73122 there was no difference observed in the abilities of palmitoyl CoA and PIP_2_ to rescue capsaicin-elicited currents (Fig. 2F).

### LC-CoA modulation of TRPV1 channel activity is side-chain length and saturation dependent

**Figure 3:**
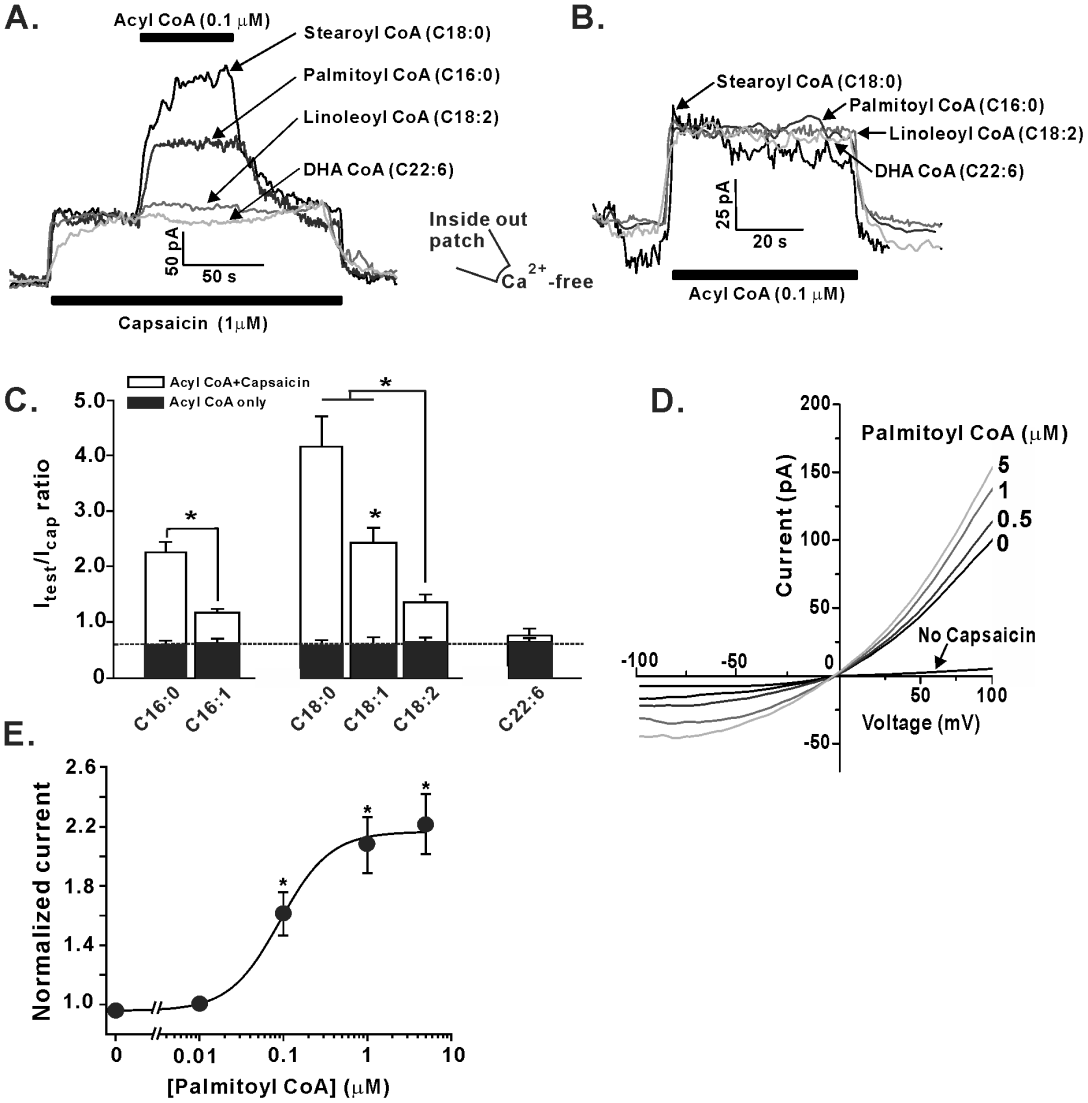
Chain-length, saturation and voltage-dependent effects of LC-CoA modulation of TRPV1 currents. (A) Representative inside-out recordings of the application various LC-CoAs (0.1 µM) in the presence, or (B) absence of 1 µM capsaicin. (C) Grouped data of the effect of 0.1 µM LC-CoAs in the presence or absence of 1 µM capsaicin. (D) I–V plot of currents elicited by 1 µM capsaicin in the presence of increasing concentration of palmitoyl CoA. (E) Concentration-effect curve of palmitoyl CoA modulation of currents elicited by 1 µM capsaicin. *P<0.05.

Previous work in our laboratory has demonstrated that LC-CoAs modulate the activity of the K_ATP_ channel and the sodium/calcium exchanger NCX1 in a side-chain length and saturation-dependent manner [23], [24]. Therefore, we hypothesized that LC-CoA side-chain length and saturation may similarly affect the interaction and activation of TRPV1 channels. Accordingly, we performed inside-out excised patch of membrane patches from tsA201 cells expressing the recombinant human TRPV1 channel (Fig. 3A). Application of LC-CoAs increased capsaicin-elicited TRPV1 current amplitudes relative to capsaicin alone (Fig. 3A). Stearoyl CoA, a saturated 18 carbon acyl CoA (C18:0), resulted in the largest increase in TRPV1 current magnitude (Fig. 3A,C). Interestingly, addition of a single or double bond in the acyl chain to generate either the monounsaturated oleoyl CoA (C18:1) or the polyunsaturated linoleoyl CoA (18∶2) reduced the stimulatory effect when compared to stearoyl CoA; with linoleoyl CoA being less stimulatory than oleoyl CoA (Fig. 3A,C). This saturation-dependent effect was also observed with LC-CoAs with a 16 carbon side-chain length; palmitoyl CoA (C16:0) and palmitoloyl CoA (C16:1) (Fig. 3C). The 22-carbon omega-3 polyunsaturated docosahexaenoic acid CoA (DHA-CoA) did not significantly augment the capsaicin-elicited TRPV1 current (Fig. 3A,C). Next, we determined the ability of LC-CoAs to stimulate TRPV1 channels in the absence of agonist. Although LC-CoAs induce small macroscopic currents in the absence of agonist (Fig. 3B), these currents were significantly smaller than those elicited by the application of 1 µM capsaicin alone and there was no difference in stimulatory effect between any of the LC-CoAs tested (Fig. 3B,C). As PIP_2_ is thought to alter the voltage-sensitivity of the TRPV1 channel (8), we investigated the voltage-dependence of LC-CoA on capsaicin-elicited TRPV1 currents effect. The addition of palmitoyl CoA increased TRPV1 currents in a concentration dependent manner, although these increases were similar for all ranges of voltage suggesting that palmitoyl CoA augmentation of TRPV1 mediated current is not voltage dependent (Fig. 3D). Finally, by adding increasing concentrations of the LC-CoA to capsaicin-elicited TRPV1 currents, we determined an EC_50_ for palmitoyl CoA of 91.4 nM (Fig 3E).

### Elevation of LC-CoAs in intact cells increases Ca^2+^ influx through TRPV1 channels

**Figure 4:**
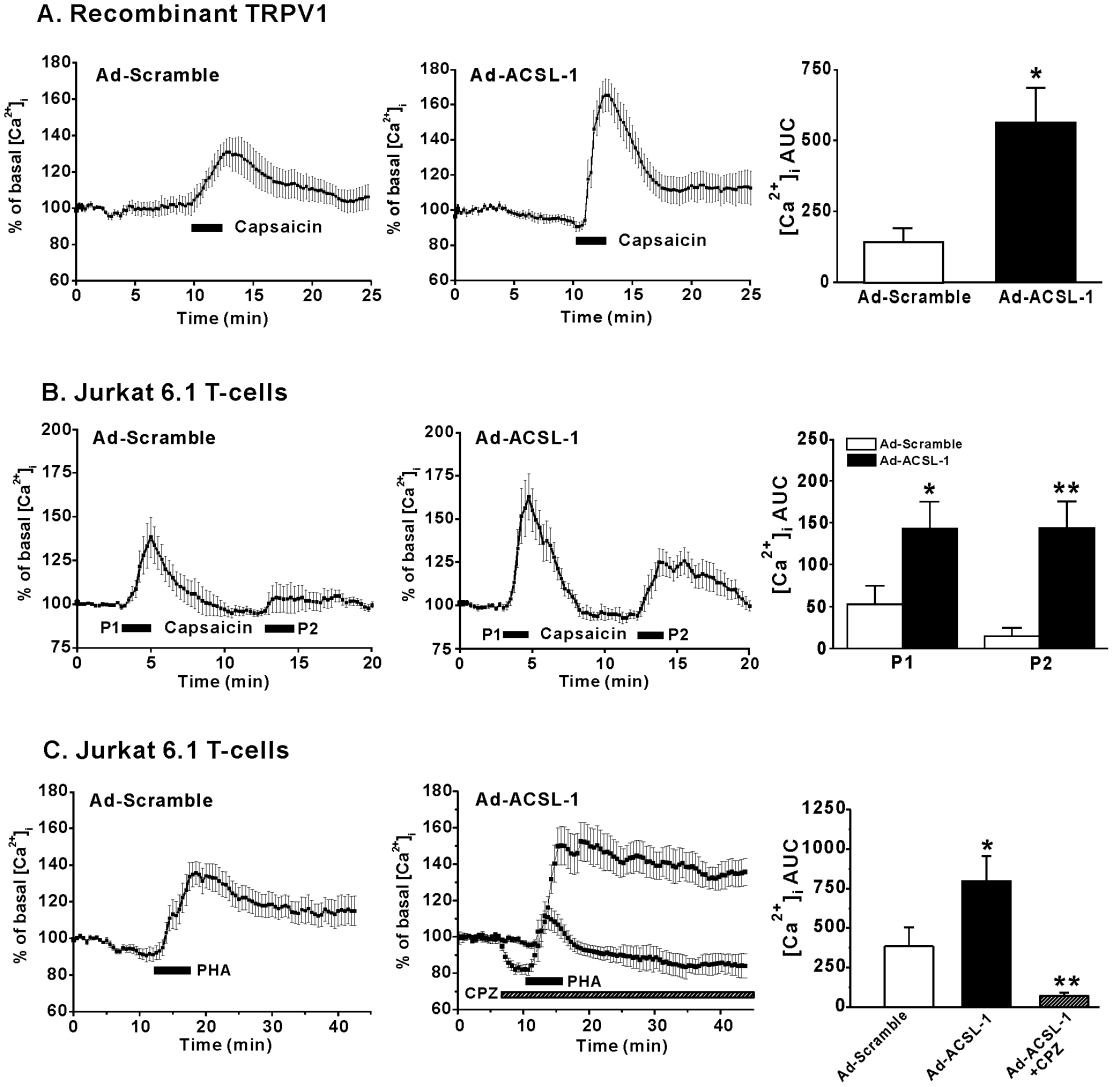
The effect of LC-CoAs on [Ca^2+^]_i_. (A) Representative intracellular paired pulse Ca^2+^ recordings and grouped data of the effect of intracellular LC-CoAs on capsaicin-elicited (1 µM) Ca^2+^ levels in tsA201 cells expressing recombinant TRPV1 channel following repeated exposure to capsaicin, Ad-Scramble (n = 5) and Ad-ACSL-1 (n = 6) or (B) Jurkat 6.1 T-cells expressing endogenous channels, Ad-Scramble (n = 8) and Ad-ACSL-1 (n = 9). (C) Representative intracellular Ca^2+^ recordings and grouped data of the effect of intracellular LC-CoAs on Jurkat 6.1 T-cells, Ad-Scramble (n = 8) and Ad-ACSL-1 (n = 9) following application of 20 µg/ml PHA. Grouped data showing the effect of 1 µM capsazepine on intracellular calcium levels in Jurkat 6.1 T-cells, Ad-ACSL-1 (n = 9) and Ad-ACSL-1+CPZ (n = 10) following application of 20 µg/ml PHA. *P<0.05, **P<0.01.

It is of importance to establish whether this observed LC-CoA-mediated increase in TRPV1 channel results in changes in intracellular Ca^2+^ in intact cells. In order to elevate endogenous intracellular LC-CoA levels in intact cells, we overexpressed LC-CoA synthetase-1 (ACSL-1) via adenoviral delivery (AdACSL-1), a manipulation we have shown previously to elevate intracellular LC-CoA levels [25]. An adenoviral vector encoding a scrambled ACSL-1 sequence served as the control (AdScramble). We first investigated whether elevation of intracellular LC-CoAs resulted in enhanced Ca^2+^ influx in tsA201 cells expressing recombinant TRPV1 channels. Following exposure to 1 µM capsaicin, control cells infected with AdScramble exhibited a moderate increase of [Ca^2+^]_i_ (142.3±48.8 AUC) (Fig. 4A). However, cells infected with AdACSL-1 displayed a significantly larger response following the application of 1 µM capsaicin, 563.2±121.4 AUC (Fig. 4A). Further experiments were performed on the human Jurkat 6.1 T-cell line to explore the effects of ACSL-1 overexpression in native immune cells with endogenous TRPV1 channel expression. We investigated the effect of repeated application of 1 µM capsaicin on the AUC of [Ca^2+^]_i_, in Jurkat 6.1 cells infected with either adenovirus. In cells infected with AdScramble the initial application of capsaicin produced a much smaller increase in [Ca^2+^]_i_ (51.4±23.1AUC) when compared to the initial increase seen in cells infected with AdACSL-1 (142.7±32.2 AUC) (Fig. 4B). In Jurkat 6.1 cells infected with AdACSL-1, although there was a decrease in peak [Ca^2+^]_i_, there was no significant decrease in the [Ca^2+^]_i_ AUC upon subsequent exposures to capsaicin (first pulse  = 142.7±32.4 AUC vs 142.7±32.4 AUC for second pulse, P>0.05, Fig. 4B). This effect was not observed in Jurkat 6.1 cells infected with AdScramble where the second pulse was less (14.7±10 AUC) than the first (51.4±23.1AUC, P = 0.005, Fig. 4B). We also investigated the effects of the known T-cell activator phytohaemagglutinin (PHA, 20 µg/ml) that is known to induce calcium influx and activate T-cells. In Jurkat 6.1 cells infected with AdACSL-1, the [Ca^2+^]_i_ AUC in response to PHA (797.0±16.0 AUC) was significantly higher than AdScramble controls (385.3±118.4 AUC) (P<0.05) (Fig. 4C). In order to show that this increase in calcium influx was mediated by the TRPV1 channel we added 1 µM of the TRPV1 antagonist capsazipine to the bath (Fig. 4C). The addition of 1 µM capsazipine decreased the PHA-induced rise in [Ca^2+^]_i_ by 90% in Jurkat 6.1 cells infected with AdACSL-1, 71.8±17.9AUC compared to 797.0±160.1AUC in its absence. These data suggest that under conditions in which intracellular LC-CoAs may be elevated [26], [27], physiological T-cell activation may be enhanced via increased TRPV1-mediated Ca^2+^ influx.

### Elucidation of residues involved in LC-CoA modulation of human TRPV1 channels

**Figure 5:**
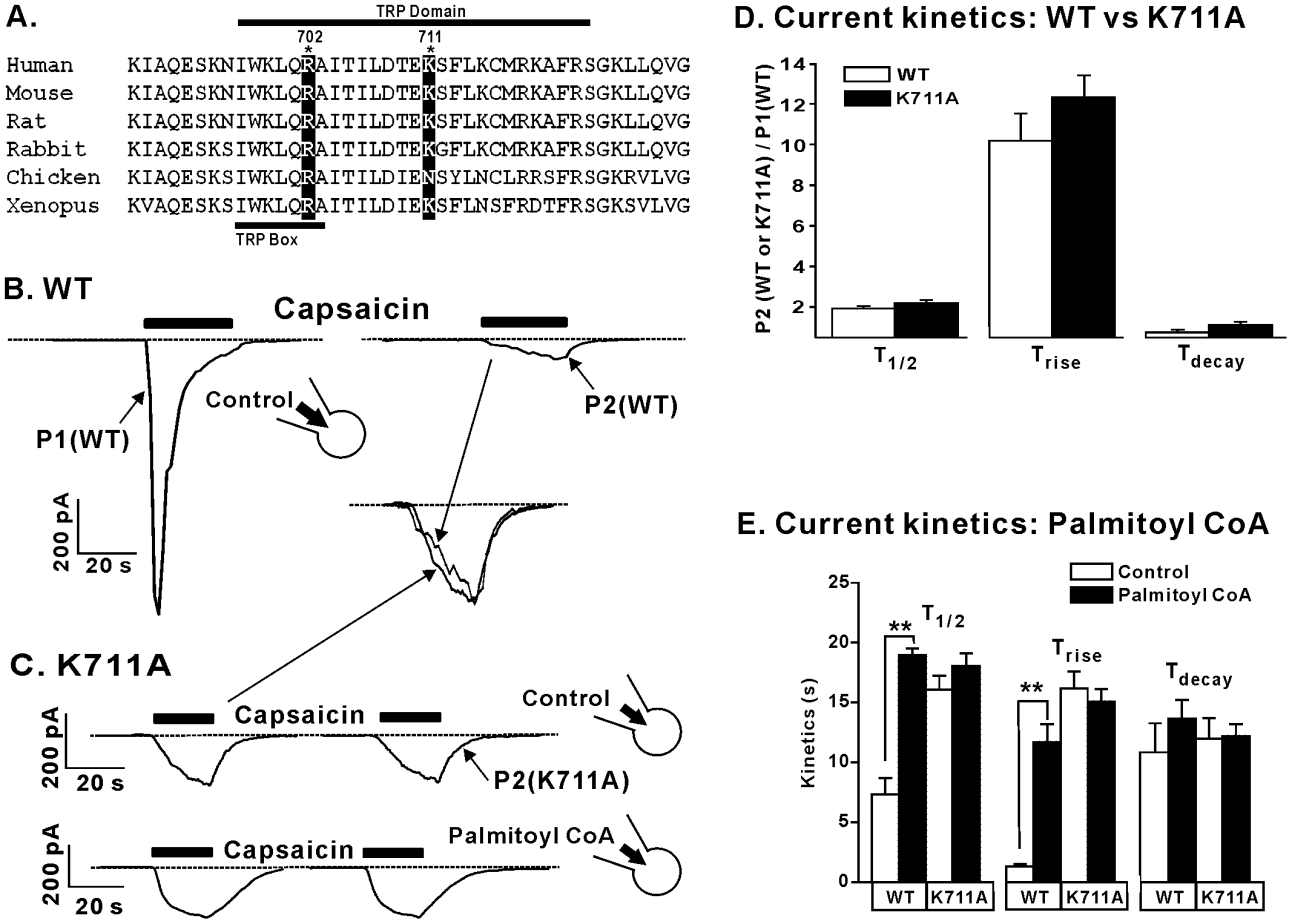
The role of K711 in LC-CoA modulation of TRPV1 channel function. (A) Amino acid sequence alignment of TRPV1. (B) Representative current trace of WT TRPV1 during repeated exposure to 1 µM capsaicin. (C) Representative current traces of the K711A TRPV1 mutant in the presence or absence of 1 µM palmitoyl CoA following repeated exposure to 1 µM capsaicin. (B,C) inset: Overlay of WT and K711A current traces normalized to maximum current illustrate the similar kinetics. (D) Grouped data of the effect of the K711A mutation on TRPV1 channel kinetics in response to 1 µM capsaicin. (E) Grouped data of the effect of 1 µM palmitoyl CoA on WT and K711A TRPV1 channel kinetics in response 1 µM capsaicin. *P<0.05, **P<0.01. Dashed line denotes zero current level.

The intracellular C-terminal region is the only cytosolic portion of amino acid sequence that is highly conserved among TRPC, TRPM and TRPV channels. The C-terminus is comprised of the TRP box that is defined by the consensus sequence of six amino acids (EWKFQR), a more variable “TRP domain” and a polybasic region that has previously been proposed to contain residues involved in PIP_2_ binding (Fig. 5A) [28]. Further studies revealed that two positively charged basic residues, R701 and K710, in the TRP domain of the rat TRPV1 sequence play an important role in PIP_2_ binding and stabilization of the PIP_2_ binding site [8]. These previous studies lead us to explore the role of these two residues (R702 and K711 in the human TRPV1 sequence) in the LC-CoA-mediated regulation of TRPV1 channels (Fig. 5A). Upon repeated exposure to 1 µM capsaicin TRPV1 currents desensitize (Fig. 5B). This phenomenon may be due to depletion of membrane PIP_2_, suggesting the reduced magnitude of currents elicited are due to TRPV1 channels in the absence of the modulatory effect of an anionic lipid moiety. To investigate the role K711 may be playing, we made the point mutation K711A and analyzed currents elicited by 1 µM capsaicin (Fig. 5C). TRPV1 K711A currents were similar in magnitude and kinetic profile in the presence and absence of 1 µM palmitoyl CoA suggesting that substitution of the basic residue K711 (Fig. 5B) leads to a loss of any potentiation if TRPV1 current by palmitoyl CoA (Fig. 5C). It is important to note that the overall macroscopic current kinetics were not significantly different between the “desensitized” wild-type TRPV1 currents and mutant TRPV1 K711A currents. Kinetic analysis shows that the T_1/2_, T_rise_ and T_decay_ of the second capsaicin application (P2) from the wild-type channel (Fig. 5B) versus currents elicited from mutant K711A channels are not significantly different, 1.9±0.1 ms vs 2.2±0.1 ms, 10.2±1.3 ms vs 12.3±1.1 ms and 0.7±0.1 ms vs 1.1±0.2 ms respectively (Fig. 5D). In addition, we were able to alter wild-type TRPV1 current kinetic parameters with the addition of 1 µM palmitoyl CoA; T_1/2_ (P<0.01) and T_rise_ (P<0.01), while addition of palmitoyl CoA had no effect of K711A mutant channels (P>0.05) (Fig. 5E and Table. 1). In contrast, the addition of palmitoyl CoA to either wild-type or the mutant TRPV1 K711A channel had no effect on T_decay_, suggesting that anionic lipids may not play a role in the “off rate” of the channel (P>0.05, Fig. 5E and Table. 1). Taken together, these data suggest that residue K711 plays a role in LC-CoA modulation of the human TRPV1 channel in a manner similar to that for PIP_2_.

**Table 1:** Kinetic parameters of WT TRPV1 and K711A mutant currents in the presence and absence of palmitoyl CoA.

	WT TRPV1			K711A	
	1^st^ current	2^nd^ current	2^nd^ current + palmitoyl CoA	Control	Palmitoyl CoA
**Peak (nA)**	−1.44±0.16	−0.22±0.08	−0.41±0.03*,**	−0.20±0.08*	−0.168±0.002*
**T1/2 (s)**	7.34±1.36	14.11±0.89	1.89±0.58*,**	16.10±1.14*	18.04±1.07*
**Trise (s)**	1.31±0.18	13.38±1.76	11.68±1.53*	16.19±1.42*	15.08±1.06*
**Tdecay (s)**	10.83±2.42	8.03±1.53	13.65±1.58*	11.98±1.73	12.20±0.97

Currents were induced by 1 mmol/l capsaicin.

* Significantly different from values in the WT control (first pulse) (P<0.05).

** Significantly different from values in the WT control (second pulse) (P<0.05).

**Figure 6:**
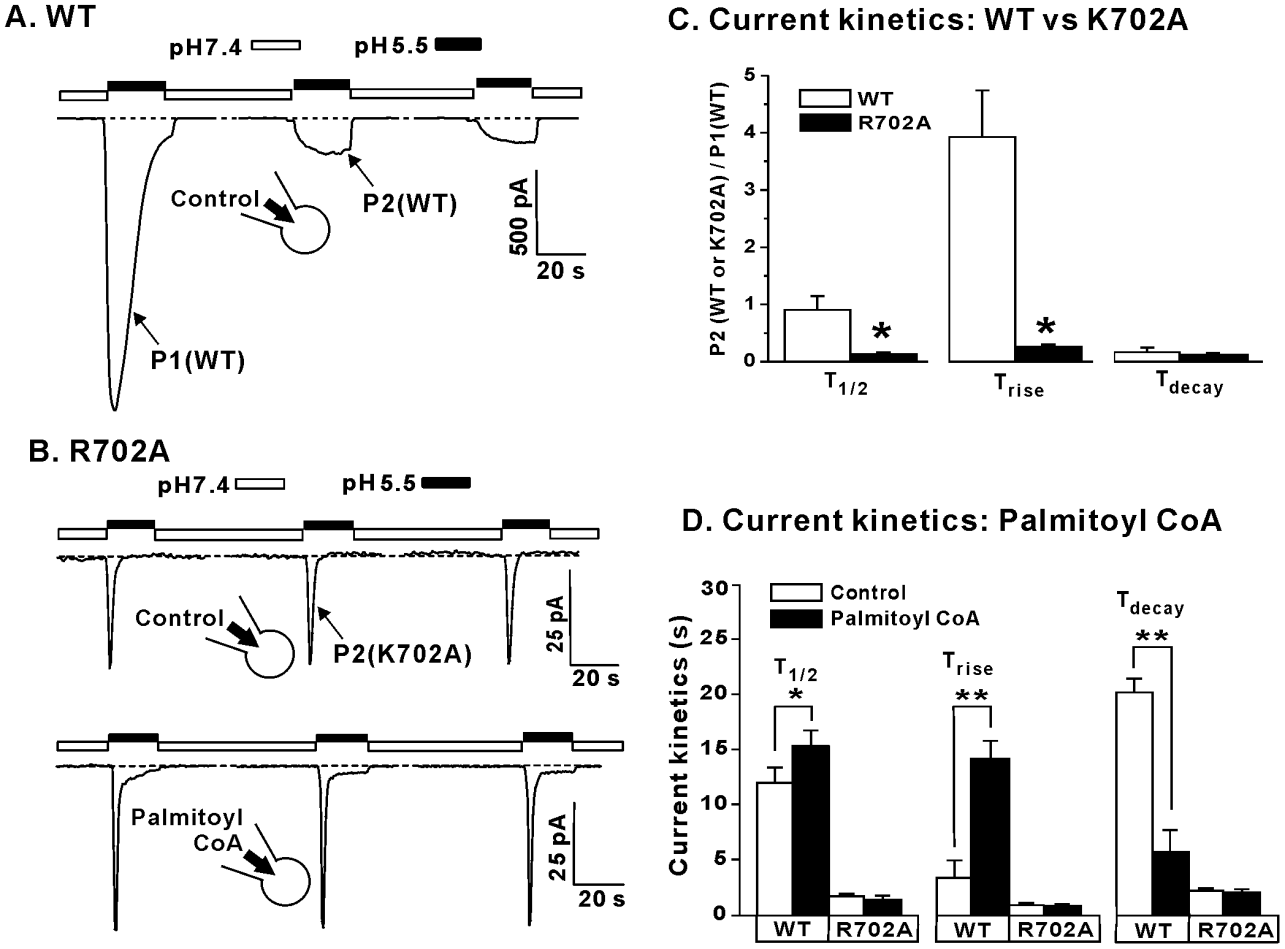
The role of R702 in LC-CoA modulation of TRPV1 channel function. (A) Representative current trace of WT TRPV1 during repeated exposure to 1 µM capsaicin. (B) Representative current traces of the R702A TRPV1 mutant in the presence or absence of 1 µM palmitoyl CoA following repeated exposure to acidic activating solution of pH 5.5. (C) Grouped data of the effect of the R702A mutation on TRPV1 channel kinetics in response to acidic solution of pH 5.5. (D) Grouped data of the effect of 1 µM palmitoyl CoA on WT and R702A TRPV1 channel kinetics in response to the pH 5.5 solution. *P<0.05, **P<0.01. Dashed line denotes zero current level.

**Figure 7:**
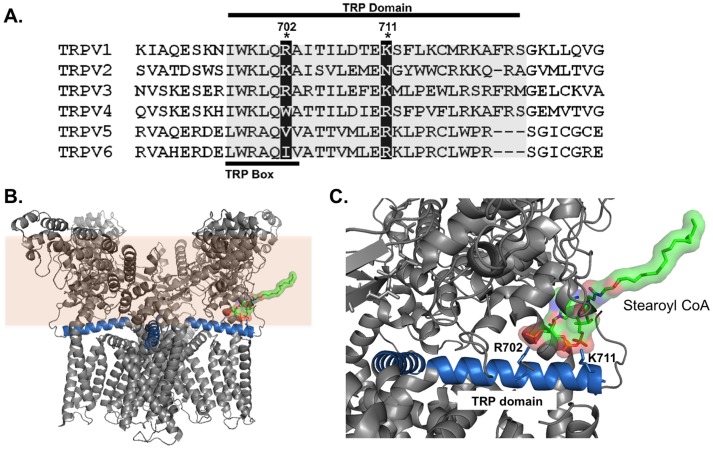
Model of LC-CoA interaction with conserved residues in human TRPV family. (A) Amino acid sequence alignment of the proximal C-terminal residues in human TRPV1-TRPV6. (B) Whole protein transmembrane view of the apo-state TRPV1 channels helical TRP domain interacting with the 18 carbon LC-CoA stearoyl CoA. (C) Synaptic view of the helical TRP domains basic residues R702 and K711 interacting with the 18 carbon LC-CoA stearoyl CoA. All molecular modeling is based on the 3.4 Å resolution TRPV1 structure determined by electron cryo-microscopy (PDB# 3J5P, [37], [38]). Analysis was performed using Pymol software.

Next we investigated the role of residue R702, homologous to R701 that has been implicated to in PIP_2_ modulation of the TRPV1 channel [8]. Interestingly, the TRPV1 R702A channel was insensitive to capsaicin (data not shown) yet it still remained responsive to acidic pH (Fig. 6A). In wild-type TRPV1, currents were desensitized in response to repeated exposure of acidic pH solution (pH 5.5) (Fig. 6A). In contrast, TRPV1 R702A currents no longer responded to the addition of 1 µM palmitoyl CoA (Fig. 6B). However, unlike the K711A mutation, the R702A mutation does not possess current kinetics similar to wild-type in the absence of PIP_2_. R702A currents had a significantly shorter T_1/2_ (P<0.05) and T_rise_ (P<0.05) while there was no change in T_decay_ (P>0.05) when compared to P2 of wild-type (Fig. 6A, C and Table 2). The T_1/2_, T_rise_ and T_decay_ of wild-type TRPV1 currents were significantly increased in the presence of 1 µM palmitoyl CoA, (P<0.01) (Fig. 6D and Table. 2). Although, addition of palmitoyl CoA had no effect in the T_1/2_, T_rise_ and T_decay_ of TRPV1 R702A currents. These data suggest that palmitoyl CoA increases the overall ionic flux in wild-type human TRPV1 channels when currents are elicited by acidic pH and that this effect is lost in the presence of the R702A mutation. In summary, both the TRPV1 K711A and R702A mutations resulted in a loss of LC-CoA modulation of the TRPV1 channel compared to wild-type currents. Interestingly, basic residues at one or both of these two positions are largely conserved throughout the entire TRPV family (Fig. 7A), suggesting that other TRPV family members may also be modulated by LC-CoAs.

**Table 2:** Kinetic parameters of WT TRPV1 and R702A mutant currents in the presence and absence of palmitoyl CoA.

	WT TRPV1			R702A	
	1^st^ current	2^nd^ current	2^nd^ current + Palmitoyl CoA	Control	Palmitoyl CoA
**Peak (nA)**	−1.50±0.35	−0.12±0.06	−0.75±0.13*,**	−0.04±0.003*	−0.06±0.004*
**T1/2 (s)**	12.02±1.32	11.02±2.7	15.33±1.36	1.61±0.91*,**	1.31±0.23*,**
**Trise (s)**	3.35±1.58	13.20±3.79	14.13±1.64*	0.86±0.06*,**	0.71±0.13*,**
**Tdecay (s)**	20.17±1.22	3.62±1.31	5.71±1.97*	2.17±0.01*	1.99±0.17*

Currents were elicited by pH5.5. * Significantly different from values in the WT control (first pulse) (P<0.05). ** Significantly different from values in the WT control (second pulse) (P<0.05).

## Discussion

Though the precise molecular mechanisms may differ, it is well known that PIP_2_ regulates the function of many trans-membrane ion transport proteins [7]. Early studies stated that PIP_2_ had a tonic inhibitory effect on TRPV1 channel function proposing that this inhibition was relieved by nerve growth factor (NGF) via activation of its tyrosine kinase receptor and effector PLC, resulting in PIP_2_ cleavage [29], [30]. Further investigation found that NGF actually led to an increase in the amount of TRPV1 channels present at the membrane, questioning the theory of PIP_2_ TRPV1 inhibition [31]. Though some controversy still exists [32] it is now generally accepted that depletion of PIP_2_ from the plasma membrane leads to inactivation of TRPV1 channels, suggesting that PIP_2_ is both a positive modulator of TRPV1 and a requirement for channel function [33]–[36]. Our current data show that TRPV1 is positively modulated by LC-CoAs. Indeed, previous work in our laboratory has shown that LC-CoAs modulate K_ATP_ channels and NCX1 in a similar manner to PIP_2_ [14], [17]. We demonstrate that LC-CoAs are potent activators of agonist-induced TRPV1 currents acting via a similar, but not identical, mechanism as PIP_2_ via interaction with the same positively charged residues.

The major differences between the effects of LC-CoAs and PIP_2_ reside in 1) the process of TRPV1 channel desensitization and 2) voltage-dependence. Upon repeated exposure to agonists such as capsaicin or acidic pH, TRPV1 channels experience almost complete desensitization. This desensitization mechanism is suggested to negatively regulate channel activity to limit excessive Ca^2+^entry. Desensitization involves Ca^2+^ dependent activation of PLC-mediated PIP_2_ depletion from the plasma membrane. Previous work has shown that the application of PIP_2_ to inside-out excised patches rescues TRPV1 from the desensitized state [35]. Our data also show that palmitoyl CoA can similarly rescue TRPV1 channels from the desensitized state following repeated application of either capsaicin or a pH 5.5 solution (Fig. 1&2). We also show that the magnitude of the PIP_2_ and LC-CoA stimulatory effect are similar and not additive, suggesting that they may be interacting at the same site(s) on the TRPV1 channel (Fig. 2D). However, unlike PIP_2_, the LC-CoA modulation of TRPV1 channels is Ca^2+^ independent (Fig. 2E). Our results also suggest that LC-CoAs are interacting with the PIP_2_ binding site in a competitive manner. Importantly, as the LC-CoA effect is Ca^2+^-independent and not susceptible to PLC-mediated cleavage, sustained increases in unbound intracellular LC-CoA levels may lead to TRPV1 channel over-activity and detrimental cellular Ca^2+^ loading.

The putative LC-CoA binding site is of obvious importance. Previous investigations have suggested that PIP_2_ interacts with residues in the C-terminus of the TRPV1 channel [8], [30]. It has previously been shown that PIP_2_ interacts with two basic amino acids in the proximal C-terminal TRP domain; R701 and K710 in the rat TRPV1 channel [8]. Recent resolution of the TRPV1 channel to 3.4 Å determined by electron cryo-microscopy suggest these charged residues in the helical TRP domain are adjacent to the cytoplasmic-membrane interface and are ideally positioned to interact with lipid modulators (Figure 7B,C) [37], [38]. In addition, the helical TRP domain interacts with the S4-S5 and S5-P-S6 domains known to be involved in channel gating and all three structures are displaced in the partially activated state when compared to the channels apo conformation [37], [38]. These data indicate that the TRP domain may be acting as a sliding helix and control gating in a similar manner to that observed in some potassium channels [39]. It has been previously proposed that the anionic head group of PIP_2_ interacts with R701 while K710 stabilizes the PIP_2_ binding region without making any direct PIP_2_ contact [8]. We therefore investigated the analogous residues in the human TRPV1 channel (R702 and K711). Our data suggests that both of these PIP_2_-interacting residues also play a role in LC-CoA modulation. As the kinetic parameters of the K711A current were indistinguishable from the WT channel in the absence of PIP_2_ (Fig. 5), we propose that K711 directly interacts with LC-CoAs. However, although the R702A mutation also resulted in a loss of the LC-CoA modulatory effect, it also resulted in significant changes in the kinetic parameters (Fig. 6). These data suggest that the R702 residue may stabilize the region where LC-CoAs interact with the channel, as mutation of this residue results in gating instability and altered channel kinetics. We propose that LC-CoAs interact with the same C-terminal basic residues as PIP_2_ (R702 and K711), likely via the negatively charged phosphate groups on the CoA moiety (Figure 7B,C). It has been previously been shown that PIP_2_ interacts with a hydrophobic pocket formed by S4–S5 linker voltage-sensing region (8). However, we did not observe any alterations to the current-voltage relationship in the presence of palmitoyl CoA (Fig. 3D), suggesting, unlike PIP_2_, LC-CoAs are not interacting with voltage-sensing domains of the TRPV1 channel.

Our data also show that LC-CoAs modulate TRPV1 channel activity in a saturation and side-chain length dependent manner (Fig. 3A–C). Increasing unsaturation decreases the magnitude of efficacy of LC-CoAs. We propose that the acyl side chain can partition into the membrane, leading to allosteric alterations in TRPV1 protein structure that result in changes in channel activity. Increasing side-chain length may strengthen the membrane partitioning of the LC-acyl tail resulting in increases in channel activity. Similarly, increasing unsaturation by the addition of double bonds would increase mobility and decrease lipophilicity of the acyl tail that may reduce acyl tail/membrane interactions and the magnitude of TRPV1 activation. This mechanism is similar to that proposed to play a role in LC-CoA activation of the K_ATP_ channel [14]. In support of this notion, it has been shown that LC-CoAs associate with membranes through insertion of the acyl side chain [40] into the bilayer, with the interaction increasing with longer side chains [41]. Furthermore, LC-CoAs may aggregate near areas of membrane curvature, such as membrane proteins, resulting higher local concentrations of LC-CoAs in the vicinity of TRPV1 channels [40]. The combination of the increased membrane interaction and decreased lateral diffusion rate of saturated and longer chain LC-CoAs may increase the longevity of TRPV1 channel opening by maintaining the CoA head group in closer contact to the basic residues identified in this study.

Intracellular LC-CoA levels are highly buffered by LC-CoA binding proteins (ACBPs), sterol carrier proteins and fatty acid bindings proteins [42]. These binding proteins are thought to be essential for correct cellular function by keeping unbound LC-CoA levels in the nanomolar range [42]–[44]. Interestingly, our finding that the palmitoyl CoA EC_50_ for the TRPV1 channel is 91 nM, suggests that the observed LC-CoA modulation of the TRPV1 channel is physiologically relevant. Furthermore, LC-CoA levels fluctuate in response to alterations in metabolic status, trans-membrane fatty acid transport and activity/expression of acyl CoA synthetases such as ACSL-1 [45]. Indeed, overexpression of ACSL-1 in either 1) cells expressing recombinant TRPV1 channels or 2) in the Jurkat6.1 T-cell line that endogenously expresses TRPV1 resulted in significantly increased [Ca^2+^]_i_ levels upon exposure to agonist (Fig. 4). These results are consistent with the concept that increasing intracellular LC-CoA levels leads to enhanced Ca^2+^ influx through TRPV1 channels.

The potential significance of our findings to health and disease still remains to be comprehensively determined in future studies. However, it is important to speculate how this LC-Acyl CoA/TRPV1 axis might contribute to both cellular function and dysfunction. Any alterations in Ca^2+^ signalling due to increased TRPV1 channel function could result in detrimental effects on a variety of cellular processes. For example, TRPV1 channel activation is thought to result in the secretion of substance P and calcitonin gene-related peptide (CGRP) that exert pro-inflammatory effects [46]. Application of capsaicin concentrations that desensitize TRPV1 channels result in a decrease in pain and levels of these inflammatory peptides in oseteoarthritis and rheumatoid arthritis [47]. TRPV1 antagonists are currently being developed to address a wide variety of diseases [48]. Interestingly, low-grade inflammation is now thought to be a central component in the development of T2D diabetes [49], a disease in which fatty acid metabolism is perturbed [50]. TRPV1 is also suggested to play a direct role in immune/inflammatory cell activation as TRPV1 channels are expressed in macrophages, dendritic cells and T-cells resulting in an increase in the levels of pro-inflammatory mediators including IL-1β, IL-6, IL-12 and TNF-α [3], [51], [52]. Therefore any mechanism that leads to excessive TRPV1 channel activity may play a role in immune/inflammatory disorders. Given the potentially important role that inflammation plays in the etiology of obesity and metabolic disorders, excessive TRPV1 activity may be involved. Indeed, exposure of rodents to capsaicin-containing diet (to desensitize TRPV1 channels) resulted in a leaner phenotype [53]. In addition, WT mice fed a high fat diet (HFD) weeks gained significantly more weight than TRPV1 knockout mice. TRPV1 knockout mice on a HFD also showed improved glucose tolerance compared to WT mice on a HFD [51], [52], [54]. Collectively these findings suggest that excessive TRPV1 activation may be a contributing mechanism to the development of obesity and T2D [51]. It is tempting to speculate that increased LC-CoA-mediated TRPV1 channel activation may occur via changes in fatty acid metabolism and transport observed in T2D and obesity where dietary consumption of saturated fatty acids is a contributory factor.

In summary, our study reports a novel mechanism by which physiological concentrations of intracellular LC-CoA potently modulate TRPV1 channel via a mechanism similar, but not identical to PIP_2_. As many TRP family members are regulated by PIP_2_, our results reveal a metabolically-linked mechanism by which TRP channels activity may be regulated. This mechanism may contribute to the cellular dysfunction observed in immune/inflammatory cell types in certain metabolic disorders that display altered fatty metabolism such as T2D and obesity. Further studies to test this concept are therefore warranted.

## Methods and Materials

### Cell culture and transfection

tsA201 cells, an SV40 transformed HEK293 cell line derivative (55), were plated on glass coverslips at low densities and allowed to grow overnight in Dulbecco's modified Eagle's medium (DMEM, Gibco) supplemented with 20 mM l-glutamine, 10% FBS and antibiotics in a humidified incubator at 37°C (5% CO2). Cells were transfected with 5 µg/dish of the human TRPV1 construct with 1 µg/dish of a plasmid encoding GFP (pGreenLantern; Life Technologies, Gaithersburg, MD) using the calcium phosphate precipitation technique (Jordan, et al., 1996). Successfully transfected cells were identified using fluorescent optics for GFP. Whole cell and macroscopic TRPV1 currents were recorded 48–72 h after transfection.

### Chemical and lipid preparation

Capsaicin (Sigma-Aldrich, St. Louis, MO) was prepared as a 1 mmol/l stock and stored at −20°C until use. Synthetic dioctanoyl (diC8)-PIP_2_ and MgATP (Sigma, Oakville, ON) were prepared as a 10 mM stock and stored at −20°C until use. The long-chain acyl CoAs palmitoyl CoA (C16:0), cis-(9)-palmitoleoyl CoA (C16:1), stearoyl CoA (C18:0), cis-(9)-oleoyl CoA (C18:1cis), cis,cis-(9,12)-linoleoyl CoA (C18:2), and all cis-docosahexaenoic acid (DHA) CoA (C22:6n-3) (Lithium salts, Sigma, Oakville, ON) and dissolved in ddH_2_O as 1 mM stock solutions. Before use, stock solutions were sonicated for 5 min and diluted in pipette solution to concentrations indicated.

### Electrophysiology

Whole-cell currents were measured using an Axopatch 200A amplifier (Axon Instruments). Patch clamp electrodes were pulled from borosilicate glass (World Precision Instruments Inc., Sarasota, FL) and fire-polished to resistances of 1–3 MΩ. The extracellular solution used to isolate TRP current contained (in mM): 135 NaCl, 5 KCl, 2 CaCl_2_, 1 MgCl2, 10 glucose, and 10 N-[2-hydroxyethyl]piperazine-N'-[2-ethanesulfonic acid] (HEPES), with pH adjusted to 7.4 using NaOH. Low extracellular Ca^2+^ solution, used to minimize TRPV1 desensitization contained 1 mM EGTA (replacing 2 mM CaCl_2_), so that Na^+^ is the major charge carrier. The pipette solution contained (in mM) 140 Cs-methanesulfonate, 2.5 NaCl, 1 MgCl_2_, 10 HEPES, 10 EGTA with pH adjusted to 7.2 using CsOH. The excised inside-out patch-clamp technique was used to measure macroscopic TRPV1 currents in transfected tsA201 cell. The solution under isometric conditions comprised (in mM): 6 NaOH, 134 NaCl, 1 mM EGTA, 1.4 MgCl_2_, 10 Glucose and 10 HEPES (pH 7.2). All drug solutions were applied to cells by local perfusion through a capillary tube positioned near the target cell. The solution flow was driven by gravity (flow rate ∼1 ml/min) and the time required to reach the cell was less than seconds. Currents were sampled using a Digidata 1322 Interface (Axon Instruments; MDS Inc. Toronto, Canada), and were low-pass filtered at 10 kz. Signals were displayed on an IBM-compatible PC using pClamp 10.0 software (Axon Instruments). Series resistance was compensated by at least 70% using the amplifier's compensation circuitry. Experiments were performed at room temperature (18–22°C).

### Ca^2+^ imaging

Ca^2+^ imaging experiments were performed as previously described using fura-2/AM (Molecular Probes, OR, USA) as the fluorescent Ca^2+^ indicator [14]. Briefly, cells cultured on poly-L-Lysine coated coverslips were loaded with 5 µM fura-2/AM and 0.02% pluronic acid for 40 min at 37°C in a balanced salt solution (containing in mmol/l: 155 NaCl, 4.5 KCl, 2 CaCl_2_, 1 MgCl_2_, 5 HEPES, and 10 glucose). Using a closed bath imaging chamber (260 µl total volume, Series 20, Warner Instruments, CT, USA), the cells were super-fused at a flow rate of 1 ml/min at 18–22°C on an inverted microscope. Fura-2 was alternately excited at 340 and 380 nm with 510 nm emission fluorescence captured and analyzed using a photo-multiplier tube system and Felix ratio-metric software (Photon Technologies International, NJ, USA).

### Site-directed Mutagenesis

The full-length human TRPV1 DNA construct was purchased from OriGene (OriGene Technologies, Rockville, MD, USA). The human TRPV1 R702A and K711A mutations were introduced using site-directed mutagenesis (QuikChange; Stratagene, CA, USA) and confirmed by sequence analysis.

### Statistical analysis

Results are expressed as mean ± SEM. Where appropriate, results were compared using 2-tailed Student's t-test (paired or non-paired) or ANOVA test. Recombinant macroscopic TRPV1 currents were normalized and expressed as an increase in current relative to capsaicin-induced control (I_test_/I_capsaicin_), where I_test_ is the current elicited by the acyl CoA stimulus and I_capsaicin_ is the current stimulated by 1 µM capsaicin. [Ca^2+^]_i_ in a treatment series were normalized with basal values obtained in the first 2 min of recording protocol (% of basal Ca^2+^).
