# Correction: Intracellular Long-Chain Acyl CoAs Activate TRPV1 Channels

**DOI:** 10.1371/journal.pone.0118385

**Published:** 2015-02-17

**Authors:** The PLOS ONE Staff

There are errors in Fig. 7. Please see the corrected Fig. 7 here:

**Fig 7:**
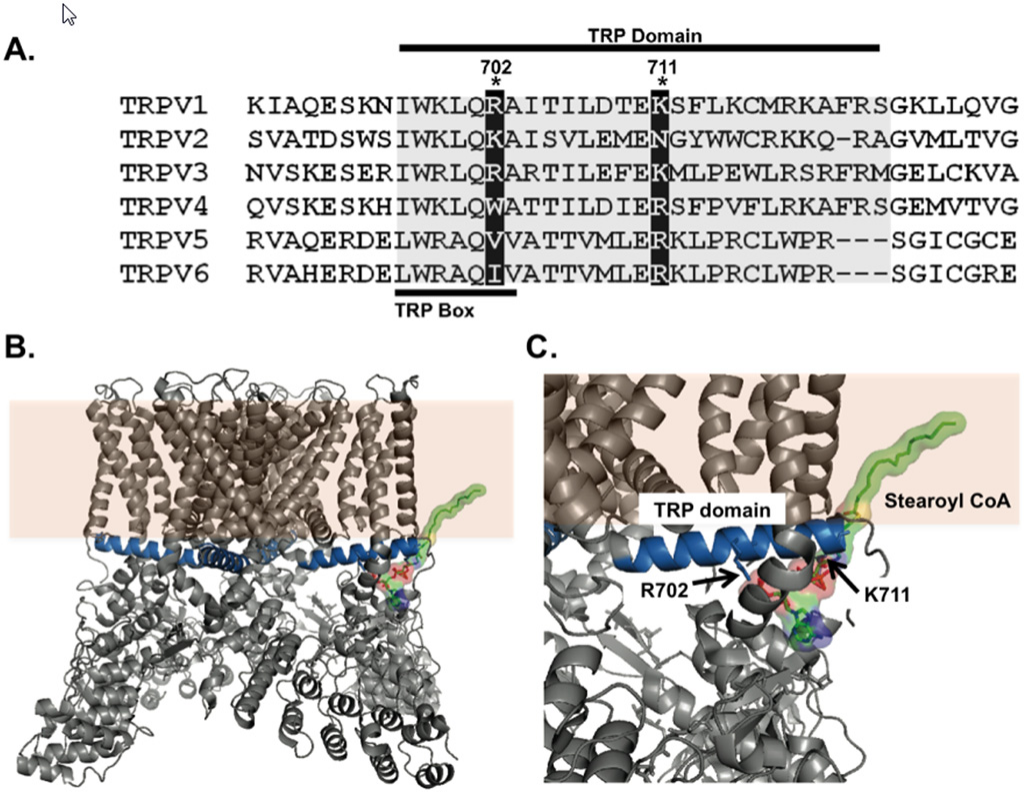
Model of LC-CoA interaction with conserved residues in human TRPV family. (A) Amino acid sequence alignment of the proximal C-terminal residues in human TRPV1-TRPV6. (B) Whole protein transmembrane view of the apo-state TRPV1 channels helical TRP domain interacting with the 18 carbon LC-CoA stearoyl CoA. (C) Synaptic view of the helical TRP domains basic residues R702 and K711 interacting with the 18 carbon LC-CoA stearoyl CoA. All molecular modeling is based on the 3.4 Å resolution TRPV1 structure determined by electron cryo-microscopy (PDB# 3J5P,[37],[38]). Analysis was performed using Pymol software.
